# Supplementary material for: Natural Killer Cells Do Not Attenuate a Mouse-Adapted SARS-CoV-2-Induced Disease in Rag2−/− Mice
Source: Viruses. 2024 Apr 15;16(4):611. doi: 10.3390/v16040611 (PMC11054502; doi:10.3390/v16040611)
Supplement: Supplementary file 1 [file viruses-16-00611-s001.zip › viruses-2865387-supplementary figures.pdf]

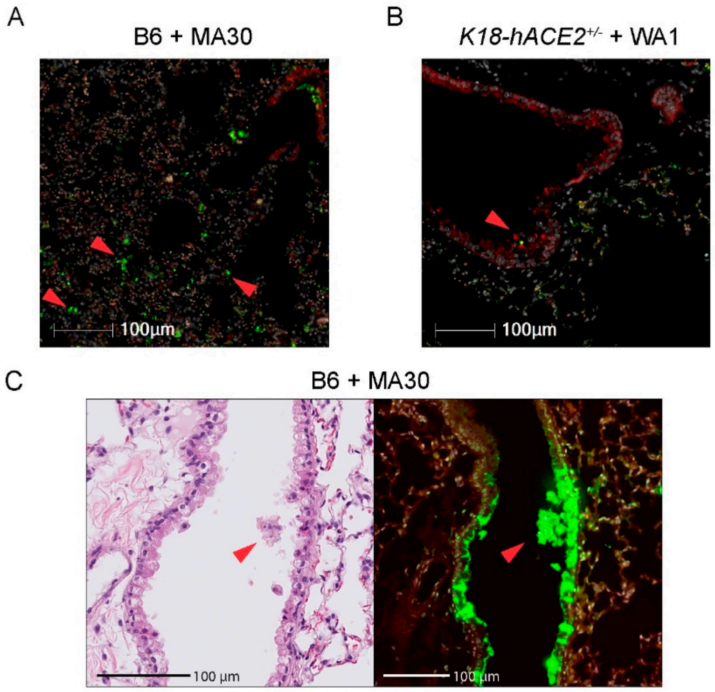

Supplemental Figure S1 SARS-CoV-2 infecting cell types and damage of the bronchial wall in the MA30-infected B6 and WA1-infected *K18-hACE2* mice. (**A**) Representative image of infection of alveolar cells (pointed by red arrows) by MA30 in B6 murine lungs at 3 DPI (**A**) and infection of peribronchial cells (pointed by red arrow) by WA1 in *K18-hACE2*$^{+/-}$ murine lungs at 3 DPI. (**C**) Images of serial sections showing sloughing of infected and necrotic epithelial cells within the bronchus in H/E (left panel, red arrow) and in immunofluorescence (right panel, red arrow) with anti-SARS in green and DAPI white.

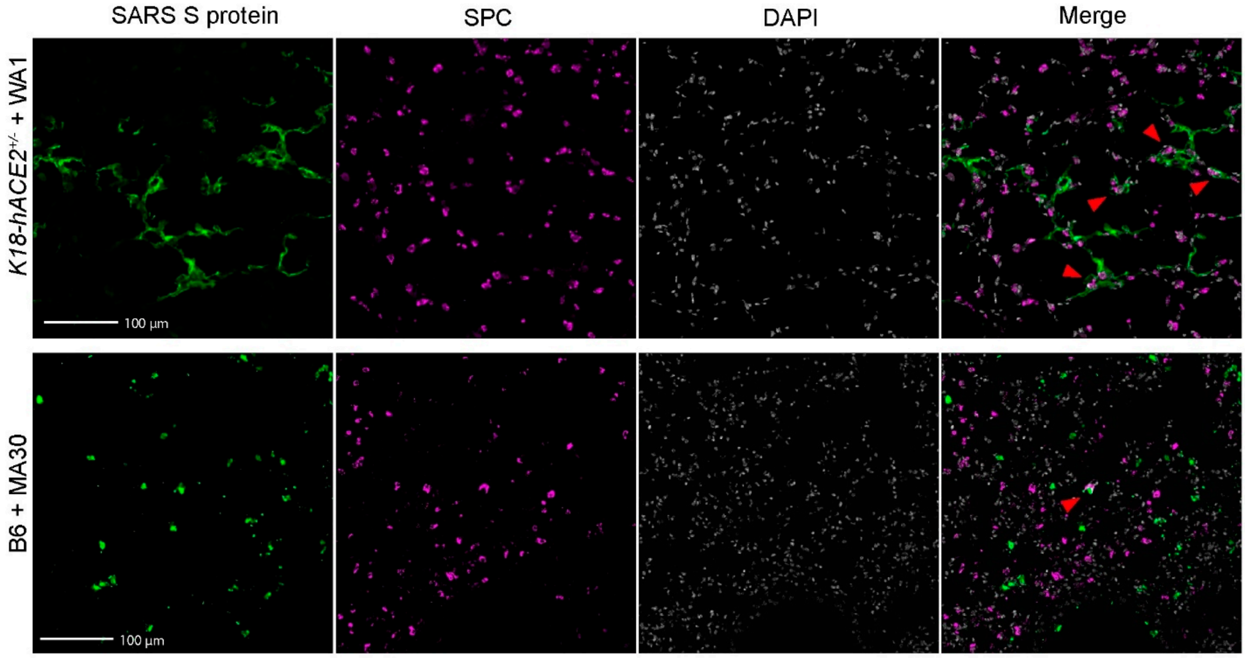

.

Supplemental Figure S2. Viral infection in AT2 cells was identified by co-staining surfactant protein C (Spc) with SARS-CoV2 spike (S) protein. Representative images from 20-week-old *K18-hACE2* (top) and B6 mice (bottom), which were intranasally infected with CoV2 MA30 (5 X 10^4 $TCID_{50}$) and CoV2 MA1 (1 X 10^4 $TCID_{50}$), respectively, and sacrificed at 3 DPI. SARS S protein in green, Spc in magenta, DAPI in white and merged channels with red arrows signaling co-localization of Spc and SARS S protein.

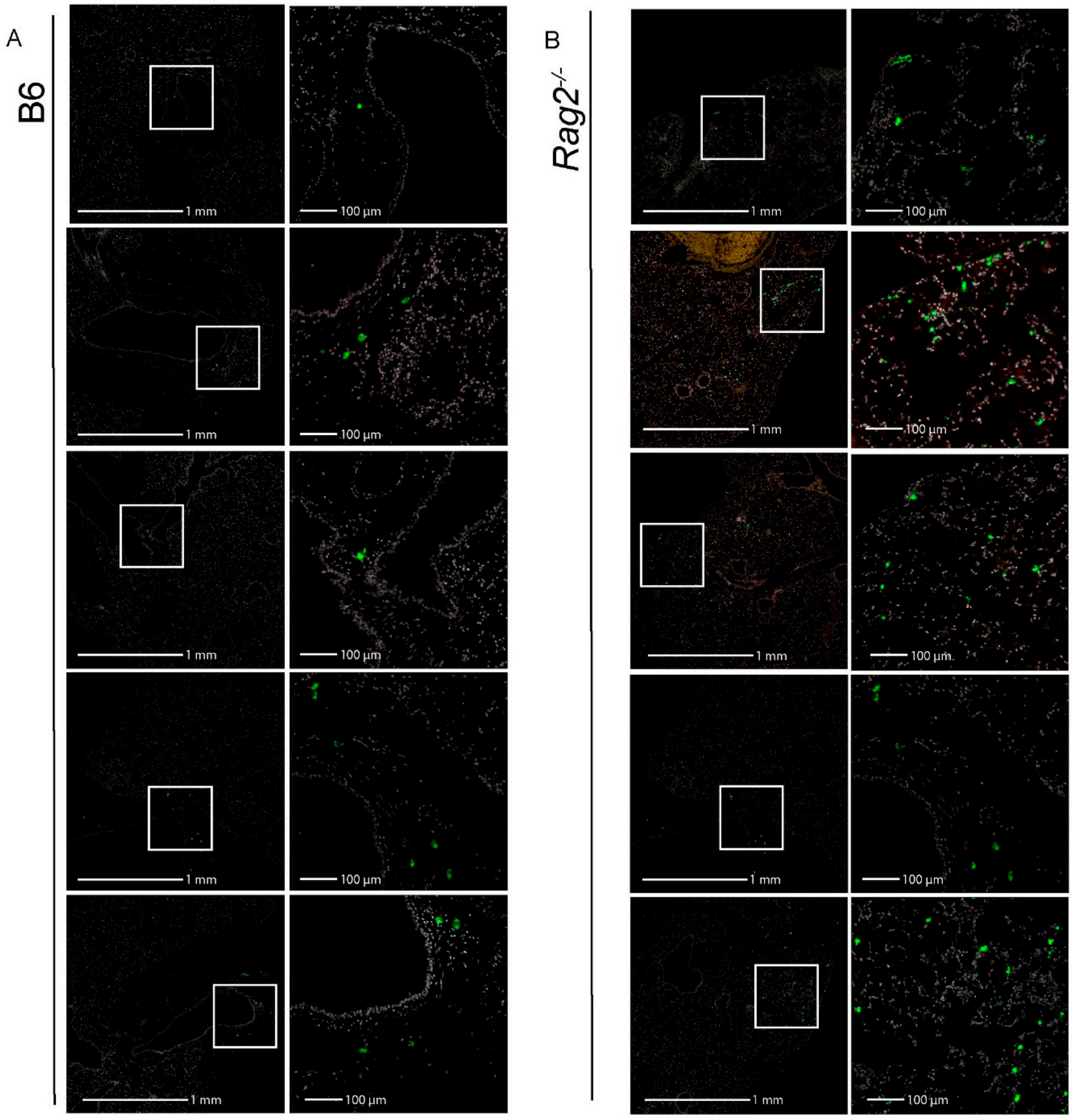

Supplemental Figure S3. Increased SARS-CoV2 Spike protein staining in lungs of the infected *Rag2*$^{-/-}$ mice. (**A**, **B**) Images of pulmonary sections for immunofluorescence staining of five different *Rag2*$^{-/-}$ mice (**A**) and five different B6 mice (**B**) infected with MA30 (5 X 10^4, $TCID_{50}$, IN) at 15 DPI. Anti-SARS S protein antibody in green and DAPI in white.

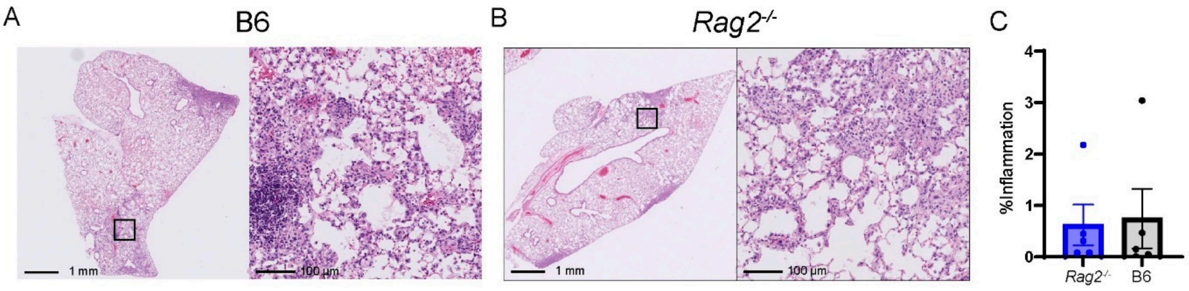

.

Supplemental Figure S4. No difference in inflammation in MA30-infected *Rag2*$^{-/-}$ and B6 mice at 15 days post infection (DPI). (A, B) Representative images of H/E stains for areas of inflammation in the infected B6 and *Rag2*$^{-/-}$ male lungs at 15 DPI (MA30, 5 X 10^4, $TCID_{50,}$ IN). (C) Quantification of B6 and *Rag2*$^{-/-}$ male mice showed no significant difference.

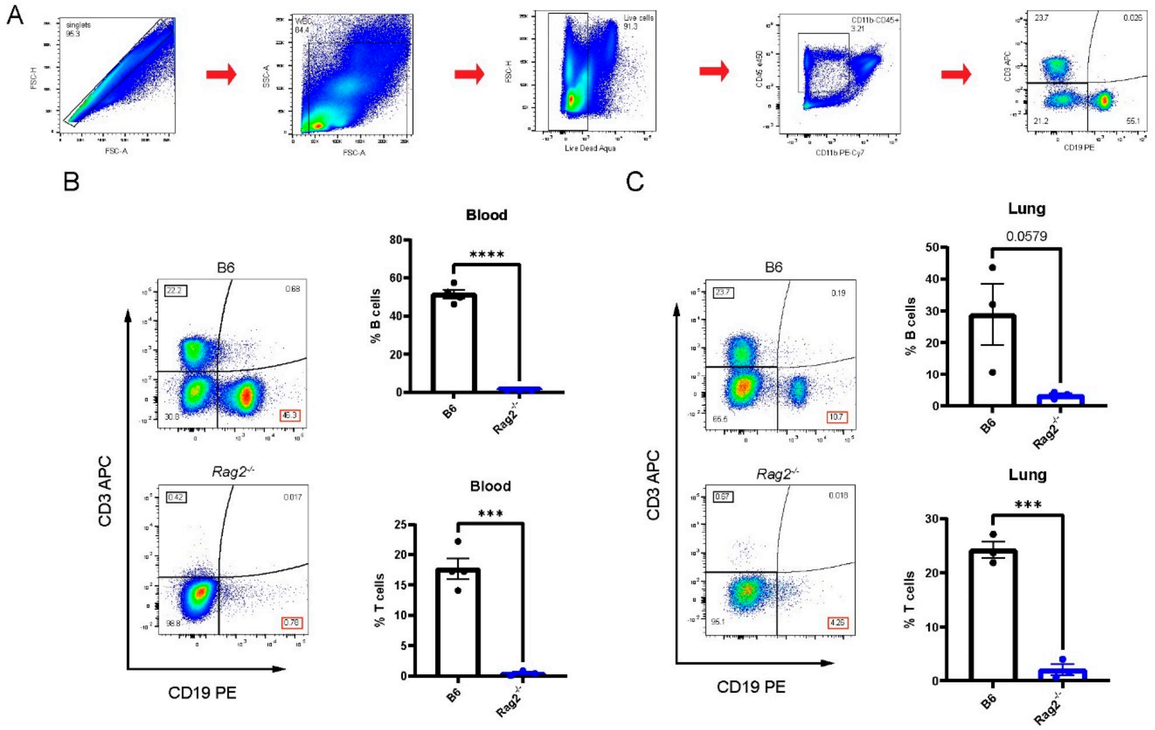

Supplemental Figure S5. Flow cytometry analysis of T and B cells from the PBMC and lung cells of B6 and *Rag2*$^{-/-}$ mice at 3 DPI (MA30, 1 X 10^4, $TCID_{50,}$ IN). (A) Gating strategy showing sequence and filtration of cells from left to right. (BC) Representative images showing percent T (black box, quadrant 1) and B cells (red box, quadrant 3) in PBMC (B) and lungs (C) of infected mice. Quantification of all mice with significant statistics shown by asterisks (****p* < 0.001; and *****p* < 0.0001) using unpaired *t*- test.

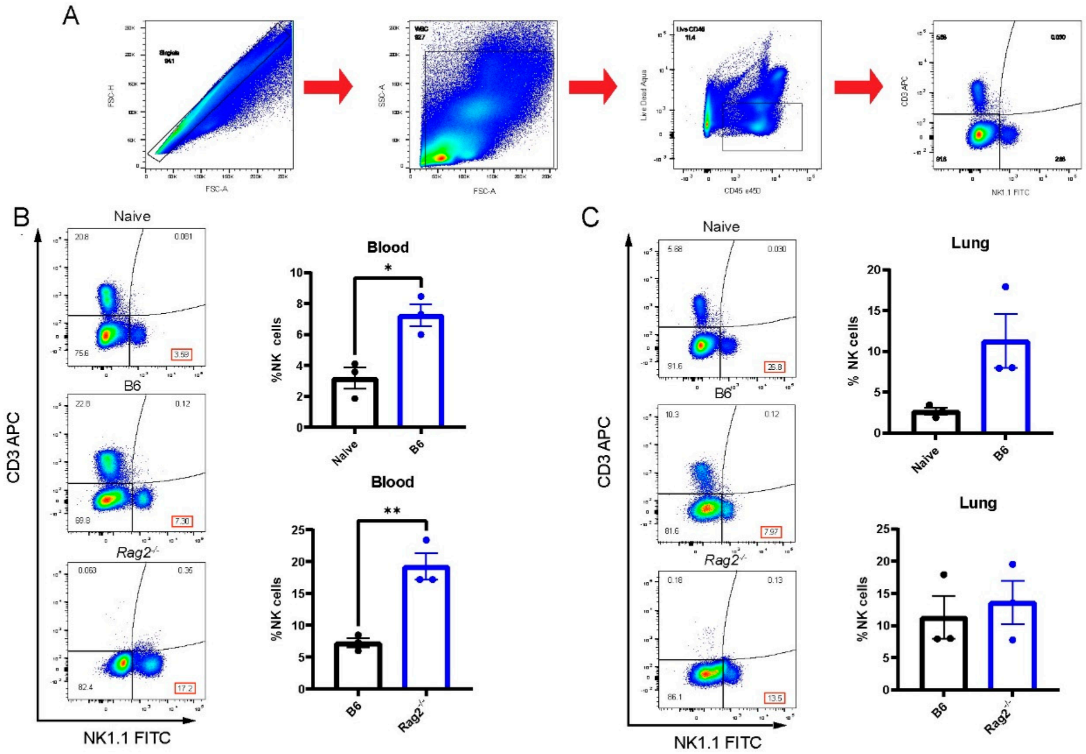

Supplemental Figure S6. Flow cytometry analysis of NK cells from the PBMC and lung cells of naive, B6 and *Rag2*$^{-/-}$ mice at 3 DPI (MA30, 1 X 10^4, $TCID_{50}$, IN). (**A**) Gating strategy showing sequence and filtration of cells from left to right. (BC) Representative images showing percent NK cells (red box, quadrant 3) in PBMC (**B**) and lungs (**C**) of naïve vsinfected mice, and infected mice vs. infected *Rag2*$^{-/-}$ *mice*. Quantification of all mice with significant statistics shown by asterisks (* p<0.05 and **p < 0.01) using unpaired t -test.

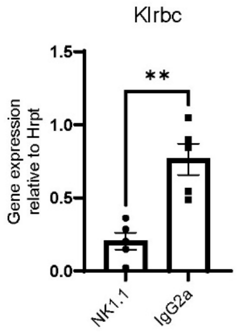

Supplemental Figure S7. Treatment of *Rag2*$^{-/-}$ mice with NK.1.1 depletes NK cells. (A) Male *Rag2*$^{-/-}$ mice depleted with anti-NK1.1 compared to isotype control IgG2a treated-mice showed significant difference (**p < 0.001 using unpaired t-test) in the lungs for Klrb1c expression levels, a gene marker for NK cell presence, normalized to Hrpt, a housekeeping gene.
